# Role of preexisting right ventricular remodeling in symptoms and prognosis after transcatheter tricuspid valve repair

**DOI:** 10.1007/s00392-024-02428-z

**Published:** 2024-03-06

**Authors:** Marc-André Ehrenfels, Caroline Fretter, Jennifer von Stein, Maria Isabel Körber, Hendrik Wienemann, Stephan Baldus, Roman Pfister, Christos Iliadis

**Affiliations:** Department for Internal Medicine III, Faculty of Medicine and University Hospital Cologne, University of Cologne, Kerpener Strasse 62, 50937 Cologne, Germany

**Keywords:** Tricuspid regurgitation, Ventricular dilation, Transcatheter tricuspid valve repair

## Abstract

**Background:**

Severe tricuspid regurgitation (TR) is associated with chronic volume overload and right ventricular remodeling (RVR). Transcatheter tricuspid valve repair (TTVr) reduces TR and can improve quality of life (QoL), but the role of preprocedural RVR on TTVr outcomes remains unclear.

**Aims:**

To investigate the role of RVR on outcomes after TTVr for severe TR.

**Methods:**

Consecutive patients undergoing TTVr (61% edge-to-edge vs. 39% direct annuloplasty) for severe TR were retrospectively compared by preexisting RVR which was defined as dilation of RV mid-level diameter (> 35 mm) according to guidelines. QoL was evaluated using NYHA class, Minnesota Living with Heart Failure Questionnaire (MLHFQ), 36-Item Short Form Health Survey (SF-36), and 6-min walking distance (6MWD) 1-month after TTVr. Mid-term mortality and heart failure (HF) hospitalization were assessed through 1 year.

**Results:**

RVR was present in 137 of 223 patients (61%). Symptoms and QoL improved equally in both groups: ≥ 1 NYHA class (57% vs. 65% of patients with vs. without RVR, respectively), 6MWD (36% vs. 34%), MLHFQ (81% vs. 69%), and SF-36 (68% vs. 65%) improvement. One-year mortality and HF hospitalization were significantly higher in patients with RVR (24% and 30%, respectively) than in patients without (8% and 13%, both *p* < 0.05). In multivariable analysis, RVR was independently associated with mortality (HR 2.3, 95%CI (1.0–5.0), *p* = 0.04) and the combined endpoint of mortality or rehospitalization (HR 2.0, 95%CI (1.1–3.8), *p* = 0.03).

**Conclusions:**

TTVr was associated with significant QoL improvement after 1 month, irrespective of RVR. Despite increased mortality and rehospitalization for heart failure, TTVr in the presence of RVR still provides substantial symptomatic benefit for patients with severe TR.

**Graphical abstract:**

Role of preexisting right ventricular remodeling (RVR) in symptoms and prognosis after transcatheter tricuspid valve repair (TTVr).

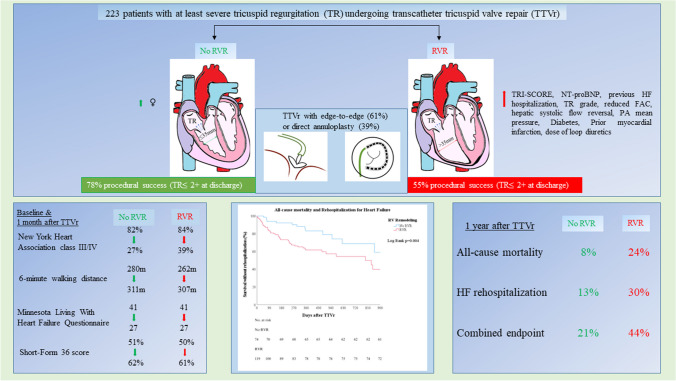

**Supplementary Information:**

The online version contains supplementary material available at 10.1007/s00392-024-02428-z.

## Introduction

Tricuspid regurgitation (TR) is one of the most prevalent valvular diseases in the elderly population and significantly worsens clinical outcomes and survival in patients with heart failure [1]. With an aging population, the prevalence of TR is expected to increase in the future [2]. Therefore, there is intensive debate about the necessity of treatment options that might impact clinical outcomes and at the same time provide sufficient safety. As conventional surgery for isolated severe TR has a significant perioperative risk and has not been proven to improve survival compared to medical management alone, treatment of symptomatic severe isolated TR has shifted towards a conservative approach for the majority of patients [3, 4]. Over the last few years, several transcatheter technologies have been developed to address the need for alternative treatment options for high-risk populations that are not eligible for surgery [5, 6]. First steps have been taken by proving the feasibility and safety of the procedure [7].

Nevertheless, the functional status of patients following transcatheter tricuspid valve repair (TTVr) regarding symptom burden and quality of life (QoL) remains poorly studied. Health-related QoL is a strong predictor of clinical outcome and all-cause mortality in patients with heart failure [8]. Hence, a major goal of TTVr treatment is to increase QoL in patients with severe symptoms [9]. It is fundamental to acknowledge TR as a heterogeneous valve disease with distinguishable phenotypes [10, 11]. Chronic volume overload occurring in severe TR culminates in right ventricular (RV) dilation and dysfunction, which has been shown to negatively affect prognosis in patients with heart failure [10, 12]. In patients undergoing isolated tricuspid valve (TV) surgery, preoperative RV remodeling (RVR) can be identified as a predictor of poor outcomes and has been associated with decreased overall survival [13, 14]. However, a systematic analysis of the TR phenotype and disease stage, which are both associated with RVR, is lacking. As TTVr is gaining importance, it is essential to evaluate the effect of preprocedural RVR in the transcatheter context. Therefore, this study aimed to analyze the impact of TTVr on procedural success, various measures of functional capacity and QoL, and mid-term clinical outcomes in the context of the presence or absence of preprocedural RVR.

## Methods

### Study population

This study was conducted at the Heart Centre at the University of Cologne. The study protocol conformed to the 1975 Declaration of Helsinki and is in line with the established Ethical Guidelines for Epidemiological Research. Consecutive patients who underwent TTVr and signed an informed consent form at our high-volume referral center were included in the study. We retrospectively studied patients between January 2017 and October 2020 and prospectively enrolled patients between October 2020 and December 2022. Despite receiving optimal medical therapy according to current guidelines, all patients were classified as New York Heart Association (NYHA) functional class ≥ II. The entire study population underwent discussion in the interdisciplinary heart team conference with a concordant decision on interventional treatment with transcatheter edge-to-edge repair or transcatheter direct annuloplasty via Cardioband implantation. This study was approved by the local ethics committee of the University of Cologne.

### Echocardiographic assessment

All patients underwent transthoracic echocardiographic assessment one day prior to the procedure. All echocardiograms were evaluated by a trained cardiologist following the current guidelines for echocardiographic assessment of valve regurgitation [15, 16]. In order to capture the severity of TR and to study the reduction in TR properly, we used a five-class grading scheme to quantify TR grade, previously proposed by Hahn and Zamorano [17]. The TR grade was classified as none or mild (I°), moderate (II°), severe (III°), massive (IV°), and torrential (V°) using qualitative (color flow jet), semiquantitative (vena contracta, systolic flow reversal in hepatic veins), and quantitative (effective regurgitant orifice area, regurgitant volume) parameters according to current guidelines. TR was secondary in all of the cases. Furthermore, right heart catheterization was performed to assess pulmonary hypertension (PH) prior TTVr. PH was classified in precapillary, isolated postcapillary, and combined pre- and postcapillary PH according to current guidelines [18]. Chronic thromboembolic pulmonary hypertension (CTEPH) was excluded via computed tomography or ventilation/perfusion scan in all patients with clinical suspicion and precapillary PH. Patients with diagnosed CTEPH were treated with specific therapy and not with TTVr and were therefore excluded from this study.

RV size was evaluated by measuring the RV cavity area and RV cavity diameter in two-dimensional RV-focused apical four-chamber view. RVR was defined as dilation of the RV at the mid-level (> 35 mm diameter, according to current guidelines) [15]. The RV basal diameter was not considered a distinct indicator of RVR, as it can be dilated along with the TV annulus in isolated RA enlargement [19]. RV dysfunction was defined as a TAPSE < 17 mm or FAC < 35%. Echocardiographic evaluation was repeated at discharge and at follow-up, approximately 4–6 weeks after the procedure.

### Evaluation of the functional capacity and QoL 

We obtained a thorough functional status for all prospective patients. Functional capacity and QoL assessments were performed by a trained medical student who was blinded to procedural and echocardiographic results. Evaluation was incorporated into the clinical routine prior to TTVr as part of the prospective analysis of our study. Six-min walking distance (6 MWD) was measured [20]. Acknowledging the occurrence of peripheral edema as a major cardinal symptom of right heart failure and TR, we quantitatively analyzed the occurrence of edema [21, 22]. The grading of peripheral edema used in this study is a modification of the classic clinical assessment described by Seidel et al. [23]. We classified three grades of edema according to severity, as measured by physical examination. Grade I was defined as pitting edema with up to 2 mm of depression and immediate rebound. Grade II was defined as pitting edema with 2–4 mm of depression and rebounding within 10–25 s. Grade III was defined as pitting edema with more than 4 mm of depression and rebound in more than 1 min. The Minnesota Living with Heart Failure Questionnaire (MLHFQ) was used to assess QoL [24]. The MLHFQ contains 21 questions (− 0–5 points for each question) representing physical, mental, and social components that can be affected by heart failure (HF). Additionally, a short version of the Short Form-36 (SF-36) health survey questionnaire containing 12 items reflecting the original 8 subscales of the SF-36 was conducted [25]. Item scores were coded, totalized, and transformed on a scale from 0 (worst health) to 100 (best health), covering functional status and general well-being as well as physical and mental health. Assessment was conducted during hospitalization before the procedure and at follow-up 4–6 weeks after the procedure.

### TTVr procedures

The procedures were performed using MitraClip, TriClip (Abbott Vascular), PASCAL P10, PASCAL Ace (Edwards Lifesciences) devices, or Cardioband device (Edwards Lifesciences), as previously described [6, 26, 27]. As direct annuloplasty is more complex than TEER, both regarding patient screening including computed tomography as well as longer and more complex procedure, TEER is the first treatment choice in daily clinical practice. In general, every patient which showed a tricuspid valve anatomy with difficulty in leaflet grasping (e.g., extremely large coaptation gap, short leaflets, or multiple-scallop leaflets) was evaluated for direct annuloplasty. Technical success was defined as successful device implantation without conversion to emergent TV surgery or re-intervention and absence of mortality, as well as successful deployment and correct positioning of the device. Additionally, procedural success was confirmed if the postprocedural TR grade at discharge was moderate or less (≤ II°).

### Endpoints

Next to TR grade, the presence and grade of peripheral edema, NYHA functional class, and QoL scores (for the prospective patient population) were assessed at 4–6 weeks following TTVr. Preprocedural RVR was assessed in all patients to analyze its impact on clinical and functional outcomes. We captured all-cause mortality, occurrence of first rehospitalization for decompensated HF, and occurrence of repeat TV intervention as mid-term clinical outcomes by contacting patients’ general physicians and using register queries.

### Statistical analysis 

Characteristics are expressed as mean ± standard deviation (SD) for continuous variables if normally distributed and as median (IQR) if not normally distributed. Normal distribution was tested for all variables using the Shapiro–Wilk test. Categorical data were presented as counts (percentages). Differences in baseline characteristics between patients with and without preprocedural RVR were determined by applying the independent *t*-test if normally distributed and the Mann–Whitney *U* test if not normally distributed for continuous variables. Differences in categorical variables were determined using Pearson’s χ^2^ test. Changes in NYHA functional class, TR grade, grade of peripheral edema, 6 MWD, and MLWFQ were studied using the Wilcoxon matched pairs signed rank test. Changes in the occurrence of peripheral edema were analyzed using the McNemar’s test. Changes in SF-36 scores were analyzed by applying a paired *t*-test. Kaplan–Meier plots were used to depict the event curves of the patients with and without RVR. Furthermore, we used Cox regression models to investigate the impact of preprocedural RVR on mid-term clinical outcomes, including all-cause mortality, HF hospitalization, and the combined endpoint. Risk was expressed as hazard ratio (HR), 95% confidence interval (CI), and *p*-value. Univariable Cox regression analysis was conducted for all baseline variables. Variables with a *p* < 0.05 in the univariable Cox regression analysis were selected for adjustment in a multivariable Cox regression model. For redundant variables, such as echocardiographic severity (VC, EROA, and RV), only one was considered for multivariable analysis. Natriuretic peptides were not included in the regression analysis because of multiple confounders (renal dysfunction and obesity). The multiparametric scores (EuroSCORE II, TRI-SCORE) were also not considered for multivariable analysis because the individual components of the scores were already included. A two-tailed *p* value < 0.05 was considered statistically significant. Statistical analyses were performed using SPSS Statistics version 28 (IBM, Armonk, NY, USA).

## Results

### Study population

A total of 223 patients who underwent TTVr were included in the study. Eighty-two patients were retrospectively analyzed, and 141 patients underwent prospective evaluation. The baseline clinical and echocardiographic characteristics are shown in Table 1. The median age was 80 years (IQR, 75–83), and 69% of the patients were female. The median European System for Cardiac Operative Risk Evaluation score II (EuroScore II) was 4.1% (IQR, 2.8–7.2%), and the mean TRI-SCORE was 6.3 ± 1.9 points. All patients had right heart failure and were symptomatic, with signs and symptoms of at least one of the following: chest discomfort, breathlessness, palpitations, and edema. Most patients were severely symptomatic at admission, with 85% presenting with NYHA functional class III or IV.

**Table 1:** Study population

	Total (*N* = 223)	RV remodeling (*N* = 137)	No RV remodeling (*N* = 86)	*P*-value (RVR vs. No RVR)
Age, y	80 (75–83)	79 (74–83)	81 (76–82)	0.177
Female	153 (69)	82 (60)	71 (83)	** < 0.001**
BMI, kg/m^2^	25.4 (22.3–29.1)	25.4 (22.7–28.9)	25.4 (21.9–29.4)	0.530
EuroSCORE II, %	4.1 (2.8–7.2)	4.5 (2.9–7.3)	3.8 (2.7–7.2)	0.163
TRI-SCORE, points	6.3 ± 1.9 ^1^	6.5 ± 1.9	6.0 ± 1.8	**0.026**
NYHA functional class				
I	4 (2)	2 (1)	2 (2)	0.677
II	30 (13)	18 (13)	12 (14)	
III	161 (72)	97 (71)	64 (74)	
IV	28 (13)	20 (15)	8 (9)	
MLHFQ, score	41 (27–52) ^2^	41 (30–54)	39 (24–49)	0.179
SF-36, %	50 ± 21 ^2^	49 ± 20	51 ± 21	0.531
6MWD, m	270 ± 106 ^3^	258 ± 111	285 ± 99	0.166
NT-proBNP, ng/L	2117 (1,344–4,129) ^4^	2627 (1,607–4,661)	1638 (1,081–2,716)	** < 0.001**
Kidney disease (GFR < 60 mL/min)	187 (84)	118 (86)	69 (80)	0.244
Increased bilirubin (> 1.2 mg/dL)	37 (17)	0.7 (0.5–1.1)	0.7 (0.5–1.0)	0.787
Clinical presentation				
Previous hospitalization due to HF	135 (60)	92 (67)	43 (50)	**0.011**
Prior peripheral edema	176 (79)	113 (82)	63 (73)	0.100
Peripheral edema at admission	129 (58)	83 (61)	46 (54)	0.296
Prior ascites	26 (12)	18 (13)	8 (9)	0.385
Echocardiographic assessment				
TR grade^5^				**0.008**
II	3 (1)	1 (1)	2 (2)	
III	126 (57)	70 (52)	56 (65)	
IV	54 (24)	32 (23)	22 (26)	
V	39 (18)	33 (24)	6 (7)	
EROA, cm^2^	0.53 (0.42–0.78) ^6^	0.62 (0.42–0.96)	0.46 (0.41–0.64)	**0.007**
VC, mm	11 (8–15) ^7^	12 (9–16)	9 (8–13)	** < 0.001**
RV, ml	46 (35–60) ^8^	50 (38–65)	39 (32–53)	** < 0.001**
TAPSE, mm	17 (14–20) ^1^	17 (14–20)	18 (15–20)	0.126
PAPsys, mmHg	43 (35–54) ^9^	43 (34–55)	43 (35–52)	0.784
RV/PA coupling, mm/mmHg	0.4 (0.3–0.5) ^10^	0.4 (0.3–0.5)	0.4 (0.3–0.5)	0.812
RV dysfunction	133 (62) ^5^	87 (66)	46 (55)	0.086
Reduced FAC (< 35%)	95 (45) ^11^	68 (53)	27 (32)	**0.003**
Hepatic systolic flow reversal	120 (74) ^12^	81 (82)	39 (62)	**0.005**
Reduced Ejection Fraction (< 50%)	42 (19) ^5^	30 (22)	12 (14)	0.133
Comorbidities				
Coronary artery disease	91 (41)	56 (41)	35 (41)	0.979
Previous CABG	20 (9)	16 (12)	4 (5)	0.074
Hypertension	184 (83)	113 (83)	71 (83)	0.988
Pulmonary hypertension	163 (73)	104 (76)	59 (69)	0.231
PAmean, mmHg	29 (24–35) ^13^	31 (24–36)	27 (24–32)	**0.049**
PAWP, mmHg	18 (14–22) ^14^	19 (16–23)	16 (14–20)	**0.006**
PVR, WU	3 (2–3) ^15^	2 (2–4)	3 (2–3)	0.693
Etiology of PH^16^		13 (16)	14 (30)	**0.036**
Precapillary PH	27 (21)60 (47)	45 (55)	15 (33)	
IpcPH	41 (32)	24 (29)	17 (37)	
CpcPH	55 (25)	43 (31)	12 (14)	
Diabetes mellitus	37 (17)	26 (19)	11 (13)	**0.003**
COPD	19 (8)	15 (11)	4 (5)	0.227
Peripheral artery disease	6 (3)	5 (4)	1 (1)	0.101
Dependent on dialysis	205 (92)	124 (91)	81 (94)	0.264
Atrial fibrillation	11 (5)	10 (7)	1 (1)	0.327
Prior myocardial infarction	26 (12)	16 (12)	10 (12)	**0.039**
Prior stroke	48 (21)	30 (22)	18 (21)	0.991
Previous heart surgery				0.864
Previous valvular intervention	40 (18)	27 (20)	13 (15)	
Mitral valve repair/replacement	10 (4)	5 (4)	5 (6)	0.384
Tricuspid valve repair	9 (4)	5 (4)	4 (5)	0.447
Cardioband	1 (0.5)	0 (0)	1 (1)	
Edge-to-edge + Cardioband				
Baseline medical treatment				
Dose of loop diuretic agents, mg/d	40 (20–100) ^5^	60 (20–100)	30 (20–52)	** < 0.001**
Sequential nephron blockade	41 (18)	29 (21)	12 (14)	0.176
Aldosterone antagonists	110 (49)	71 (52)	39 (45)	0.346
ACE inhibitors/ARBs	91 (41)	57 (42)	34 (39)	0.759
Beta-blockers	191 (86)	117 (85)	74 (86)	0.894
Cardiac devices				
Pacemaker	49 (22)	35 (25)	14 (16)	0.104
ICD	4 (2)	4 (3)	0 (0)	0.110
CRT	7 (3)	6 (4)	1 (1)	0.180

Values are median (IQR), *n* (%), or mean ± SD. Pulmonary hypertension was defined as mean pulmonary artery pressure > 20 mmHg. Precapillary pulmonary hypertension was defined as PAmean > 20 mmHg and PAWP ≤ 15 mmHg and PVR > 2 WU. IpcPH was defined as PAmean > 20 mmHg and PAWP > 15 mmHg and PVR ≤ 2 WU. CpcPH was defined as PAmean > 20 mmHg and PAWP > 15 mmHg and PVR > 2 WU. Right ventricular remodeling was defined as RV diameter > 35 mm at mid-level. RV dysfunction was defined as FAC < 35% or TAPSE < 17 mm

^1^*N* = 219, ^2^*N* = 132, ^3^*N* = 114, ^4^*N* = 214, ^5^*N* = 222, ^6^*N* = 164, ^7^*N* = 215, ^8^*N* = 151, ^9^*N* = 210, ^10^*N* = 209, ^11^*N* = 211, ^12^*N* = 162, ^13^*N* = 163, ^14^*N* = 157, ^15^*N* = 146, ^16^*N* = 128

*6MWD*, 6-min walk distance; *ACE*, angiotensin-converting enzyme; *ARB*, angiotensin receptor blocker; *BMI*, body mass index; *CABG*, coronary artery bypass grafting; *COPD*, chronic obstructive pulmonary disease; *CpcPH*, combined post- and precapillary pulmonary hypertension; *CRT*, cardiac resynchronization therapy; *eGFR*, estimated glomerular filtration rate; *EROA*, effective regurgitant orifice area; *EuroSCORE*, European System for Cardiac Operative Risk Evaluation; FAC, fractional area change; HF, heart failure; ICD, implantable cardioverter defibrillator; IpcH, isolated postcapillary pulmonary hypertension; *NT-proBNP*, N-terminal pro-B-type natriuretic peptide; *NYHA*, New York Heart Association; *PA*, pulmonary artery; *PAmean*, mean pulmonary artery pressure; *PAPsys*, systolic pulmonary artery pressure; *PH*, pulmonary hypertension; *RV*, regurgitant volume; *TAPSE*, tricuspid annular plane systolic excursion; *TR*, tricuspid regurgitation; *VC*, vena contracta

*p*-values < 0.05 are shown in boldface

A total of 137 patients (61%) had preprocedural RVR, and 86 patients (39%) had no RVR. Patients with RVR were significantly more male (*p* < 0.001). Both groups were highly symptomatic, with a similar distribution of NYHA functional class grading. Patients with RVR received a significantly (*p* < 0.001) higher dose of loop diuretics at baseline (60 mg/day, IQR, 20 mg/day to 100 mg/day with RVR, vs. 30 mg/day, IQR, 20 mg/day to 50 mg/day without RVR; *p* < 0.001) than patients without RVR. The occurrence of peripheral edema did not differ between the groups. Prior to TTVr, patients with RVR had more frequently a history of hospitalization for HF (67% vs. 50%, *p* = 0.01), diabetes mellitus (31% vs. 14% of patients, *p* = 0.003), prior myocardial infarction (7% vs. 1%, *p* = 0.039), higher NT-proBNP level (median 2,627 vs. 1,368 ng/l, *p* < 0.001), and higher TRI-SCORE (6.5 ± 1.9 vs. 6.0 ± 1.8, *p* = 0.026). A detailed comparison of the baseline characteristics of the patients with and without RVR is presented in Table 1.

### Echocardiographic assessment

The TR grade was significantly higher in patients with RVR compared with those without RVR. Forty-seven percent vs. 33% of patients with vs. without RVR, respectively, presented a TR grade of massive or torrential (*p* = 0.008). Patients with RVR also showed higher values for all parameters of quantitative TR grade measurement (EROA 0.62 vs. 0.46 cm^2^, *p* = 0.007; RV 50 vs. 39 ml, *p* < 0.001; VC 12 vs. 9 mm, *p* < 0.001). The frequency of pulmonary hypertension did not differ among the groups (76% vs. 69%, *p* = 0.231). The mean pulmonary artery pressure measured in right heart catheterization was higher in patients with RVR (31 vs. 27 mmHg, *p* = 0.049). Additionally, the mean pulmonary artery wedge pressure was higher in patients with RVR (19 vs. 16 mmHg, *p* = 0.006). The etiology of pulmonary hypertension showed marked differences between the two groups, with patients with RVR having a distribution of 16% with precapillary pulmonary hypertension, 55% with isolated postcapillary hypertension, and 29% with combined precapillary and postcapillary hypertension, whereas those without RVR had percentages of 30%, 33%, and 37%, respectively, for the aforementioned classifications (*p* = 0.036). Whereas the mean TAPSE was not statistically different among groups (17 mm vs. 18 mm, *p* = 0.126), reduced FAC (< 35%) was more frequent in patients with RVR (68% vs. 27%, *p* < 0.001). Hepatic systolic flow reversal was more frequent in patients with RVR (82% vs. 62%, *p* = 0.005). A detailed comparison of the baseline echocardiographic assessments of the patients with and without RVR is presented in Table 1.

### Procedural results of TTVr

Cardioband implantation was performed in 87 patients (39%), and MitraClip in the tricuspid position was used in 11 patients (5%). TriClip devices were used in 49 patients (22%). PASCAL devices were used in 76 patients (34%). Technical success was achieved in 209 (94%) patients. In seven patients (3%), TTVr was unsuccessful due to unfavorable anatomical, technical, or procedural circumstances, and the device was removed without complications. Procedural success, defined as a moderate or less TR at hospital discharge, was achieved in 64% of the attempted procedures. None of the patients was lost to follow-up at 1 month regarding vital status. Seven patients (3%) died postprocedural or during the 1-month follow-up period.

There was no difference regarding the presence of RVR at baseline between patients undergoing edge-to-edge repair and patients undergoing direct annuloplasty (59% vs. 65%, *p* = 0.316). The technical success rate was higher in patients without RVR (98% vs. 91% with RVR, *p* = 0.054). All-cause mortality rate within 1 month of TTVr was higher in patients with RVR (*p* = 0.033). Procedural success rate was higher in the absence of RVR (78% vs. 55%, *p* < 0.001). There were no differences in procedural aspects or complications between TTVr devices. Head-to-head comparison of procedural information and major complications associated with the two different TTVr strategies are shown in the supplemental file. A detailed comparison of procedural results is presented in Table 2. TR grade at discharge was significantly lower in patients without RVR (*p* = 0.004). In comparison with TR grade at baseline, the TR grade at 1-month follow-up was significantly reduced regardless of RVR. In comparison with TR grade at discharge, the TR grade at the 1-month follow-up did not change in either group. Fifty-six percent vs. 78% of patients with vs. without RVR presented with a NYHA class of ≤ II at discharge (*p* = 0.004).

**Table 2:** Procedural aspects of TTVr

	Total (*N* = 223)	RV remodeling (*N* = 137)	No RV remodeling (*N* = 86)	*P*-value (RVR vs. No RVR)
Technical success	209 (94)	125 (91)	84 (98)	0.054
Procedural success	143 (64)	76 (55)	67 (78)	** < 0.001**
TR grade at discharge				
I	58 (26)	28 (20)	30 (35)	**0.004**
II	86 (39)	49 (36)	37 (43)	
III	62 (28)	44 (32)	18 (21)	
IV	12 (5)	11 (8)	1 (1)	
V	5 (2)	5 (4)	0 (0)	

Values are *n* (%). Technical success was defined as successful device implantation without conversion to emergent TV surgery or re-intervention, absence of mortality, and successful deployment and correct positioning of the device. Procedural success was defined as postprocedural TR grade at discharge was moderate or less (≤ II)

*p*-values < 0.05 are shown in boldface

### Functional capacity and QoL

Overall, at the 1-month follow-up, the rate of NYHA functional class III/IV reduced from 83% at baseline to 34% at the 1-month follow-up (*p* < 0.001). Grade III peripheral edema decreased from 10% at baseline to 4% at the 1-month follow-up (*p* = 0.003). The mean 6 MWD increased from baseline to 1-month follow-up (*p* < 0.001), with 35% of patients showing a clinically relevant improvement of 50 m or more. The median MLHFQ score decreased from baseline to 1-month follow-up (*p* < 0.001), with 76% of the patients presenting with a clinically relevant improvement of at least 5 points. Additionally, the mean SF-36 score increased from baseline 1-month follow-up (*p* < 0.001), with 67% of patients showing a clinically relevant improvement of at least 2.5 points.

Significant improvement in NYHA functional class was observed in patients regardless of RVR. At the 1-month follow-up, NYHA functional class improved by at least one grade in 57% and 65% of the patients with and without RVR, respectively (*p* = 0.262). The rate of NYHA functional class III/IV decreased from 84 to 39% vs. from 82 to 27%, at the 1-month follow-up in patients with and without RVR, respectively (*p* < 0.001).

**Fig. 1:**
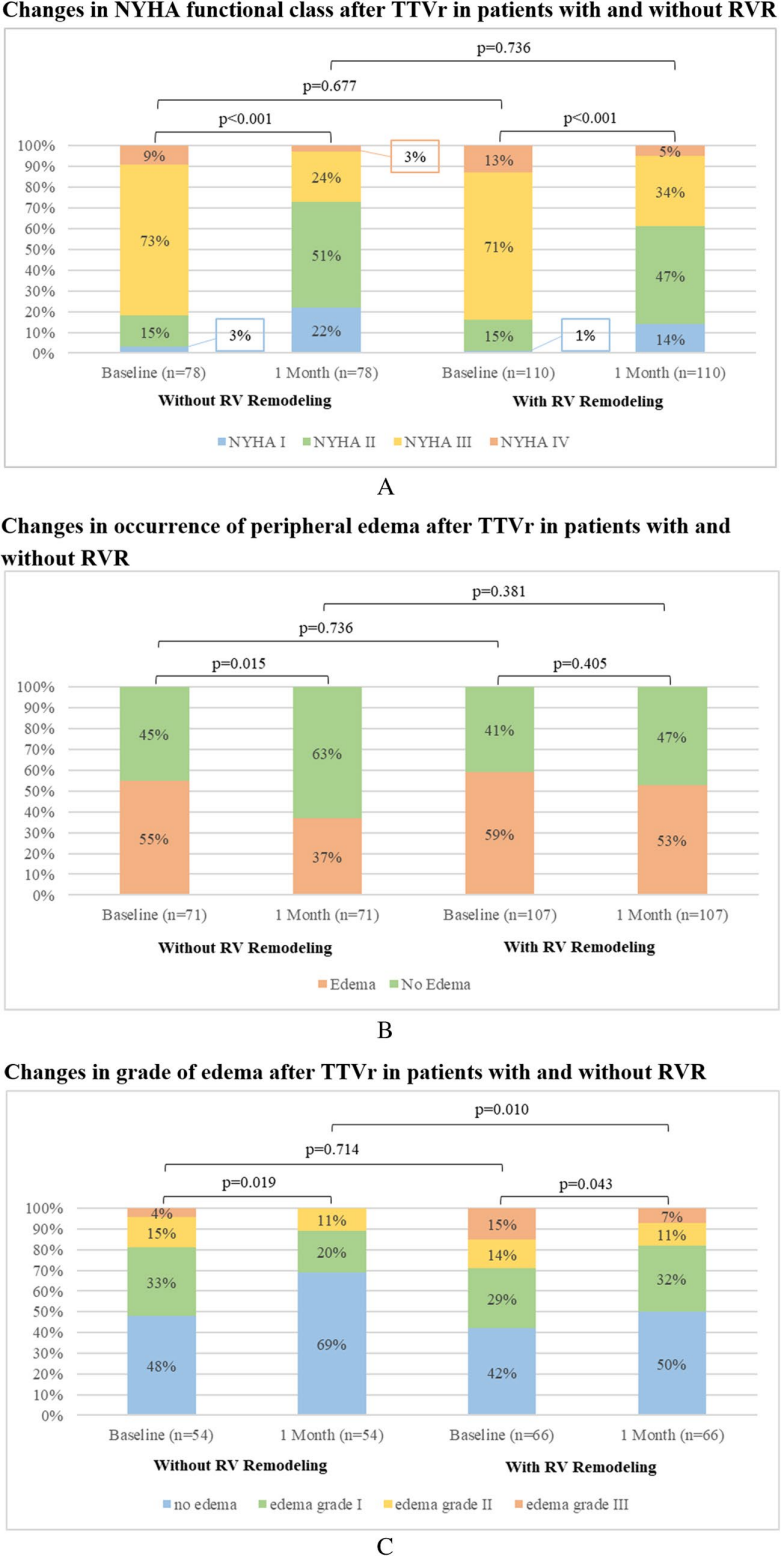
**A** Comparison of 1-month changes in NYHA functional class after TTVr in patients with and without RVR. **B** Comparison of 1-month changes in the occurrence of peripheral edema after TTVr in patients with and without RVR. **C** Comparison of 1-month changes in grade of peripheral edema after TTVr in patients with and without RVR

The occurrence of peripheral edema decreased from 59 to 53% vs. from 55 to 37% at the 1-month follow-up in patients with and without RVR, respectively. A significant reduction in the occurrence of peripheral edema was observed only in patients without RVR (*p* = 0.015). Patients with and without RVR showed a significant decrease in the grade of peripheral edema, with a decrease of at least one grade in 30% of patients with RVR and 31% of patients without RVR. Changes in NYHA class and the status of peripheral edema are illustrated in Fig. 1.

**Fig. 2:**
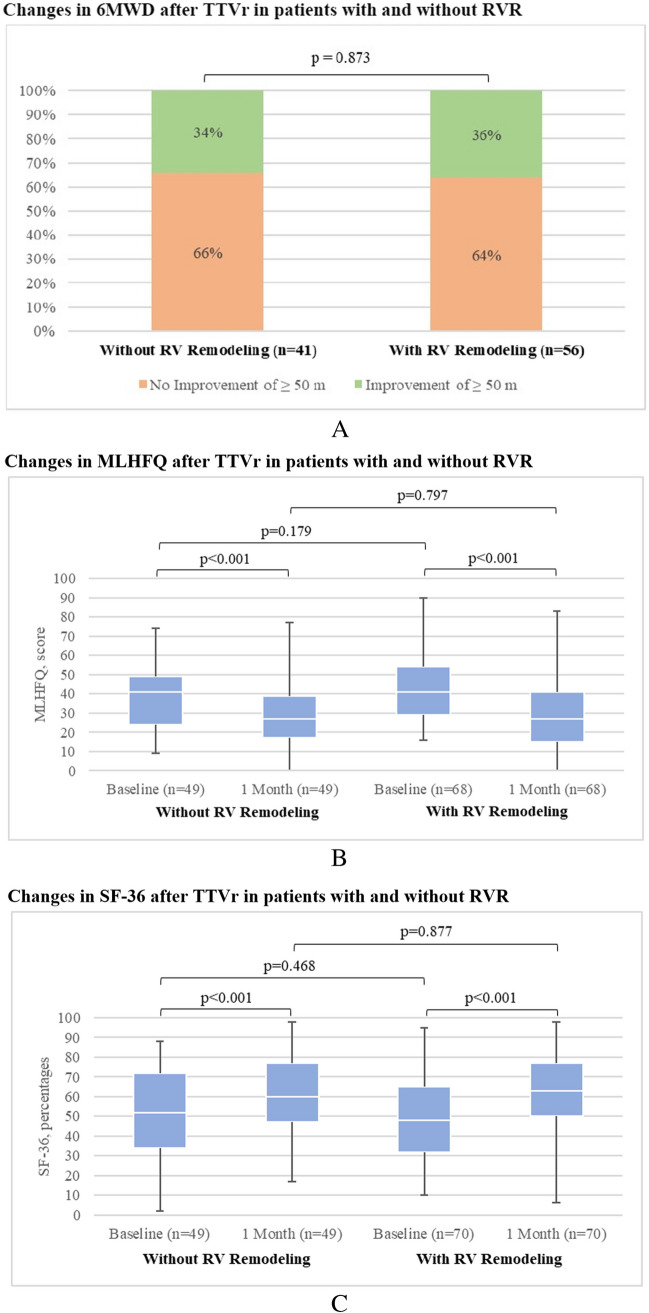
**A** Comparison of 1-month changes in 6MWD after TTVr in patients with and without RVR. **B** Comparison of 1-month changes in MLHFQ after TTVr in patients with and without RVR. **C** Comparison of 1-month changes in SF-36 after TTVr in patients with and without RVR

In patients with RVR, the mean 6 MWD increased from 262 m ± 109 at baseline to 307 m ± 110 at the 1-month follow-up (*p* < 0.001), whereas in patients without RVR, the mean 6 MWD increased from 280 m ± 104 to 311 m ± 98 at the 1-month follow-up (*p* = 0.016). Thirty-six percent and 34% showed a clinically relevant increase in the 6 MWD of at least 50 m for patients with and without RVR, respectively, as shown in Fig. 2.

Both groups showed significant improvement in QoL at the 1-month follow-up compared with the preprocedural baseline. In patients without RVR, the median MLHFQ score decreased from 41 (IQR, 24–49) at baseline to 27 (IQR, 17–39) at the 1-month follow-up (*p* < 0.001), and 69% showed an improvement of at least 5 points. In comparison, the mean MLHFQ score in patients with RVR decreased from 41 (IQR, 29–54) at baseline to 27 (IQR, 15–41) at the 1-month follow-up (*p* < 0.001), and 81% showed an improvement of at least 5 points. Additionally, the mean SF-36 score in patients without RVR improved from 51 ± 22% at baseline to 62 ± 19% at the 1-month follow-up (*p* < 0.001), with 65% of the patients showing an improvement of at least 2.5 points. The mean SF-36 score in patients with RVR increased from 50 ± 21% at baseline to 61 ± 21% at the 1-month follow-up (*p* < 0.001), with 68% of patients showing an improvement of at least 2.5 points. Functional capacity and QoL did not differ between devices. The changes in the MLHFQ and SF-36 scores are shown in Fig. 2.

QoL data were available for 35 patients at 12 months. NYHA functional class was available for 59 patients at 12 months. 6MWD was available for 30 patients at 12 months. Median MLHFQ scores at 12 months were 21 (IQR, 11–39) and 32 (IQR, 19–37) in patients with and without RVR, respectively. The mean SF-36 scores at 12 months were 65 ± 20% and 53 ± 19% in patients with and without RVR, respectively.

The rate of NYHA functional class III/IV at 12 months was 36% and 32% in patients with and without RVR, respectively. The incidence of peripheral edema at 12 months was 33% and 30% in patients with and without RVR, respectively. The mean 6MWD at 12 months was 324 ± 148 and 309 ± 108 in patients with and without RVR, respectively.

### Mid-term clinical outcomes

Mid-term follow-up data were available after 463 ± 403 days (median, 374 days; IQR, 156–607). Six-month follow-up was available in 84% of patients, and 12-month follow-up was available in 68% of patients. Information about death after TTVr was available in all patients, and information about rehospitalization due to HF after TTVr was available in 76% of patients.

One year after the procedure, 19% of the patients died, 25% were readmitted to the hospital due to decompensated heart failure, and one patient had to undergo repeat TTVr because of severe residual TR.

**Fig. 3:**
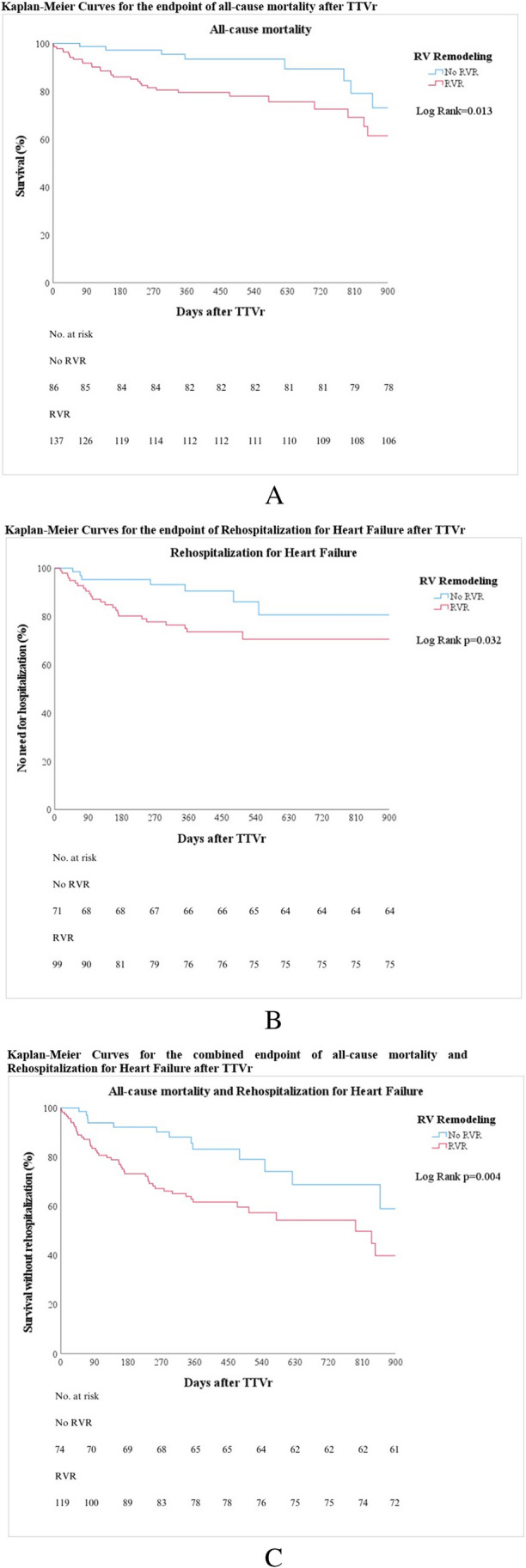
**A** Kaplan–Meier curves for the endpoint of all-cause mortality after TTVr. **B** Kaplan–Meier curves for the endpoint of rehospitalization for heart failure after TTVr. **C** Kaplan–Meier Curves for the combined endpoint of all-cause mortality and rehospitalization for heart failure after TTVr

All-cause mortality 1 year after TTVr was significantly higher in patients with RVR (24% vs. 8%, *p* = 0.013), and RVR was associated with a HR of 2.6 (95% CI, 1.2–5.6) for all-cause mortality (*p* = 0.016). One-year heart failure (HF) rehospitalization after TTVr was significantly higher in patients with RVR (30% vs. 13%, *p* = 0.045), and RVR was associated with a HR of 2.4 (95% CI, 1.1–5.7) for rehospitalization due to HF after TTVr (*p* = 0.038). The combined endpoint of 1-year mortality or 1-year rehospitalization due to HF after TTVr was seen significantly more often in patients with RVR (44% vs. 21%, *p* = 0.012), and RVR was associated with a HR of 2.4 (95% CI, 1.3–4.5) for the combined endpoint of mortality or rehospitalization due to HF after TTVr (*p* = 0.005). Outcomes between devices were not different. The need for repeat TTVr was not associated with RVR. The Kaplan–Meier curves of all-cause mortality and rehospitalization due to HF are shown in Fig. 3.

In the multivariable analysis, RVR remained to be independently associated with mid-term mortality (HR 2.3, 95%CI (1.0–5.0), *p* = 0.042) and the combined endpoint (HR 2.0, 95%CI (1.0–3.8), *p* = 0.027). Mid-term clinical outcomes did not differ between devices. A detailed comparison of the uni- and multivariable analysis is presented in Table 3.

**Table 3:** Cox regression analysis for the endpoints of all-cause death, heart failure hospitalization (HFH), and the combination of both endpoints

		Univariable analysis		Multivariable analysis	
		Hazard ratio (95% CI)	*p*-value	Hazard ratio (95%CI)	*p*-value
Right ventricular remodeling	Death	2.6 (1.2–5.6)	**0.016**	2.3 (1.0–5.0)	**0.042**
	HFH	2.4 (1.1–5.7)	**0.038**	1.9 (0.8–4.6)	0.136
	Combination	2.4 (1.3–4.5)	**0.005**	2.0 (1.1–3.8)	**0.027**
Age (years)	Death	1.0 (0.9–1.0)	0.108		
	HFH	1.0 (0.9–1.0)	0.674		
	Combination	1.0 (0.9–1.0)	0.380		
Female sex	Death	0.5 (0.3–1.0)	0.052		
	HFH	0.6 (0.3–1.1)	0.211		
	Combination	0.7 (0.4–1.1)	0.133		
Procedural success	Death	0.5 (0.3–0.9)	**0.023**	0.6 (0.3–1.2)	0.146
	HFH	0.5 (0.3–0.9)	**0.042**	0.6 (0.3–1.3)	0.218
	Combination	0.4 (0.2–0.7)	** < 0.001**	0.5 (0.3–0.9)	**0.015**
BMI (kg/m^2^)	Death	0.9 (0.9–1.0)	0.294		
	HFH	1.0 (0.9–1.1)	0.432		
	Combination	1.0 (0.9–1.1)	0.584		
NYHA class III or IV	Death	5.0 (0.7–36.9)	0.111		
	HFH	1.8 (0.6–6.0)	0.326		
	Combination	2.3 (0.8–6.5)	0.101		
eGFR < 60 (ml/min)	Death	1.6 (0.6–4.0)	0.354		
	HFH	1.9 (0.6–6.1)	0.310		
	Combination	1.3 (0.6–2.7)	0.531		
Bilirubine > 1.2 (mg/dl)	Death	2.6 (1.3–5.0)	**0.005**	2.4 (1.2–4.7)	**0.010**
	HFH	1.5 (0.6–3.6)	0.401		
	Combination	2.0 (1.1–3.5)	**0.021**	2.3 (1.2–4.2)	**0.007**
6MWD (m)	Death	1.0 (0.9–1.0)	0.138		
	HFH	1.0 (0.9–1.0)	0.180		
	Combination	1.0 (0.9–1.0)	0.050		
Reduced RV function	Death	2.1 (0.9–4.5)	0.068		
	HFH	2.5 (1.1–5.7)	**0.036**	1.9 (0.8–4.6)	0.161
	Combination	2.2 (1.2–4.0)	**0.011**	1.7 (0.2–3.2)	0.105
Vena contracta mm	Death	1.0 (0.9–1.1)	0.974		
	HFH	1.0 (0.9–1.1)	0.667		
	Combination	1.0 (0.9–1.1)	0.830		
EF < 50 (%)	Death	1.4 (0.7–2.9)	0.334		
	HFH	3.7 (1.8–7.5)	** < 0.001**	2.4 (1.1–5.4)	**0.034**
	Combination	2.2 (1.3–3.8)	**0.004**	1.6 (0.9–3.1)	0.128
Coronary artery disease	Death	1.1 (0.6–2.0)	0.849		
	HFH	2.1 (1.0–4.2)	**0.049**	1.4 (0.6–3.2)	0.395
	Combination	1.5 (0.9–2.6)	0.098		
Previous CABG	Death	1.7 (0.7–4.0)	0.239		
	HFH	2.8 (1.1–6.8)	**0.025**	1.0 (0.3–2.8)	0.980
	Combination	2.6 (1.4–5.1)	**0.004**	1.5 (0.5–4.3)	0.419
Arterial hypertension	Death	0.7 (0.4–1.6)	0.441		
	HFH	2.0 (0.6–6.7)	0.242		
	Combination	1.2 (0.6–2.3)	0.675		
Pulmonary hypertension	Death	0.8 (0.4–1.7)	0.609		
	HFH	2.4 (0.8–6.8)	0.107		
	Combination	1.6 (0.8–3.0)	0.163		
Leaflet repair vs. annuloplasty	Death	1.4 (0.7–2.7)	0.294		
	HFH	1.0 (0.5–2.1)	0.896		
	Combination	1.1 (0.7–1.9)	0.629		
Diabetes mellitus	Death	1.7 (0.9–3.3)	0.121		
	HFH	0.9 (0.4–2.1)	0.791		
	Combination	1.2 (0.7–2.2)	0.476		
COPD	Death	0.4 (0.7–3.1)	0.380		
	HFH	2.0 (0.9–4.4)	0.073		
	Combination	1.5 (0.8–2.9)	0.168		
Peripheral artery disease	Death	1.3 (0.5–3.6)	0.651		
	HFH	1.1 (0.3–3.7)	0.863		
	Combination	1.1 (0.5–2.7)	0.762		
Atrial fibrillation	Death	0.7 (0.2–2.0)	0.491		
	HFH	1.0 (0.2–4.0)	0.953		
	Combination	0.7 (0.3–1.8)	0.485		
Prior myocardial infarction	Death	0.5 (0.1–3.6)	0.492		
	HFH	0.8 (0.1–5.6)	0.784		
	Combination	0.7 (0.2–3.0)	0.666		
Prior stroke	Death	1.2 (0.5–2.8)	0.699		
	HFH	1.5 (0.6–3.6)	0.770		
	Combination	1.1 (0.5–2.2)	0.835		
Previous heart surgery	Death	1.6 (0.8–3.1)	0.168		
	HFH	1.4 (0.6–3.1)	0.421		
	Combination	1.8 (1.0–3.0)	**0.044**	1.0 (0.4–2.1)	0.923

Pulmonary hypertension was defined as mean pulmonary artery pressure > 20 mmHg. Right ventricular remodeling was defined as RV diameter > 35 mm at mid-level. RV dysfunction was defined as FAC < 35% or TAPSE < 17 mm. Procedural success was defined as postprocedural TR grade at discharge was moderate or less (≤ II). Leaflet repair was defined as implantation of a TriClip, MitraClip, or PASCAL. Annuloplasty was defined as implantation of a Cardioband

*6MWD*, 6-min walk distance; *BMI*, body mass index; *CABG*, coronary artery bypass graft; *COPD*, chronic obstructive pulmonary disease; *EF*, ejection fraction; *eGFR*, estimated glomerular filtration rate; *NYHA*, New York Heart Association

*p*-values < 0.05 are shown in boldface

## Discussion

This study analyzed procedural success, functional capacity, and QoL, as well as mid-term clinical outcomes in patients with symptomatic HF due to TR following TTVr regarding preprocedural RVR. The main findings can be summarized as follows: (i) TTVr is a safe and effective treatment option in patients regardless of RVR; (ii) TTVr resulted in significant QoL improvement at 1 month; (iii) patients with RVR showed higher 1-year mortality and HF hospitalization; and RVR was independently associated with all-cause mortality and the combined endpoint of mortality or rehospitalization after TTVr.

### Importance of preprocedural RV remodeling in TTVr

It is of fundamental importance to acknowledge functional TR as a heterogeneous valve disease with distinguishable phenotypes regarding its pathophysiology [10, 11]. The two main phenotypes of functional TR that must be differentiated are atrial functional TR, in which the development of regurgitation is primarily due to right atrial dilation, and ventricular functional TR, in which the main mechanism leading to regurgitation is dilation and remodeling of the right ventricle with consequent tethering of valve leaflets [11, 28]. However, in the literature, the definitions of atrial TR vary. Previous definitions of atrial TR mainly based on clinical criteria, whereas there is currently a trend of using echocardiographic parameters to classify TR etiology [1, 11, 29]. For reasons of clinical utilization and with respect to the primary pathophysiological processes, we decided to approximate the entity of atrial TR as TR in the absence of RVR, as proposed by Prihadi et al. in 2019 [30]. Consequently, RVR was defined as dilation of the RV at the mid-level (> 35 mm diameter, according to current guidelines) [15]. A thorough multiparametric and multimodal investigation of the heterogeneous TR population is sophisticated and cumbersome to apply on a daily basis. To this end, the definition was based on only one variable to provide a simple and hands-on parameter for risk assessment of patients undergoing TTVr in everyday clinical practice. The RV basal diameter was not considered a distinct indicator of RVR, as it can be dilated along with the TV annulus in isolated RA enlargement [19].

As patients with progressive RVR showed significant functional abnormalities, these patients had more frequently reduced FAC (< 35%) (68% vs. 27%, *p* < 0.001). The role of RVR on outcomes of patients undergoing treatment of TR is largely unknown. TR grade at baseline was significantly higher in patients with RVR than in those without RVR (*p* = 0.008). Nevertheless, we acknowledge the relationship between RVR and severity of TR as part of a continuous vicious cycle in which causality cannot be completely clarified, since RVR influences TR severity and vice versa [31]. However, the manifestation of RVR is an important finding that shows an advanced and/or adverse clinical stage in patients with TR. Considering surgery as the only treatment of TR until recently, Calafiore et al. reported an HR of 6.47 (95% CI, 3.88–10.77) for patients with RVR who underwent isolated TV surgery (vs. HR of 2.6 in our study), which confirmed that RVR is a risk factor for lower survival [13]. In this study, RVR was defined as either RV dilation (basal end-diastolic diameter > 42 mm and/or mid-level diameter > 35 mm) or dysfunction (tricuspid annular plane systolic excursion < 16 mm and/or tissue Doppler S-velocity < 10 cm/s) [13]. Consequently, the comparability of the results with those of our study may be limited because of differences in the definition of RVR. However, the patient population with RVR defined by our approach (dilation of RV at mid-level with diameter > 35 mm) was included in their definition of RVR. The fact that RVR has a larger impact on outcomes after surgery compared to TTVr has numerous explanations. Because right heart failure is accelerated by myocardial ischemia after cardiopulmonary bypass and suboptimal myocardial protection during surgery, the increased strain on the right heart during cardiac surgery compared with TTVr might be an important reason for worse surgical outcomes in patients with preexisting RVR [32]. Another contributing factor of worsening right heart function with cardiac surgery might be the need for excessive blood transfusions and the subsequent increase in right ventricular preload, which further worsens right ventricular function with RVR [32]. Therefore, the reasons mentioned above might be seen as advocacy for the transcatheter approach in patients with RVR, even though RVR is still associated with an increase in mortality compared with patients without RVR.

### Technical and procedural success of TTVr

The present study confirmed that TTVr is a safe procedure in a highly symptomatic patient population, which showed a substantial surgical risk profile and was therefore not eligible to undergo a surgical approach for TV repair. The rate of technical success we observed was high and comparable to that of recent studies conducted in a similar patient population undergoing TTVr [33]. High technical success rates were observed in both surveyed phenotypes of TR, and no significant differences were observed between patients with and without preprocedural RVR. Interestingly, the vast majority of unsuccessful device implantations occurred in patients with RVR.

The TR degree was significantly reduced after TTVr in patients with and without prior RVR at the 1-month follow-up. The prevalence of procedural success was significantly higher in patients without RVR than in those with RVR (55% vs. 78%,* p* < 0.001). This is an important finding which could be explained by greater degree of TR and TV deformation including larger valve diameter with larger coaptation gap due to RV dilation and consecutively more challenging anatomy for catheter-based treatments.

In comparison to the considerably less complex mitral valve anatomy, Yoshida et al. reported no differences in procedural success and residual regurgitation between patients with atrial functional MR and those with ventricular MR undergoing percutaneous edge-to-edge mitral valve repair [34]. Nevertheless, we acknowledge MR and TR as separate valve diseases that differ in etiology and clinical impact on heart failure progression. Therefore, the results of TMVr regarding ventricular remodeling are not directly transferrable to TTVr and are comparable only to a limited extent.

### Functional capacity and QoL

The present study captured functional capacity and QoL by analyzing NYHA functional class, occurrence, and grade of peripheral edema, and 6 MWD, as well as changes in MLHFQ and SF-36 scores. There was a significant improvement in NYHA functional class at the 1-month follow-up compared with baseline, with 66% of patients having NYHA class I or II, which is promising. Smaller series have shown previously that NYHA class can be reduced after TTVr, albeit with off-label use of edge-to-edge device [35]. Here, we did not see a device-related difference in outcomes. Additionally, there was a significant improvement in both surveyed phenotypes, which might indicate a favorable short-term functional outcome of TTVr, regardless of prior RVR or even reversibility of RVR by TTVr. As peripheral edema is a cardinal symptom of TR, we observed changes in the occurrence and grade of edema before and after TTVr. We observed a significant improvement in peripheral edema grade from baseline to 1-month follow-up in both groups, which might imply that even though peripheral edema was still present in some patients, right heart function improved, and volume overload decreased after TTVr in the majority of cases. This is further affirmed by the fact that no up-titration in loop diuretics doses was observed at the 1-month follow-up compared with baseline; therefore, we cannot attribute the edema regression to escalated medical therapy. Nevertheless, peripheral edema was completely absent in 47% and 63% of 1-month follow-up for patients with and without RVR, respectively (*p* = 0.029), which might indicate that RVR has a negative impact on right heart function after TTVr. Moreover, we assessed functional capacity by comparing the 6 MWD in all prospectively recruited patients. Overall improvement of 6 MWD was + 39 m (significantly higher compared with baseline, *p* < 0.001). Small reports on edge-to-edge devices only showed a mean 6 MWD increase of approximately 33 m [36]. However, a clinically relevant increase of at least 50 m was present in only one-third of patients and was not different between groups. Furthermore, the present study demonstrated that TTVr improved QoL by showing significant improvement in MLHFQ and SF-36 scores at the 1-month follow-up, which is comparable to previous studies investigating QoL after TTVr and TMVr, respectively [33, 37]. In addition, we could complement the analysis by showing that there is an improvement in QoL after TTVr regardless of RVR presence.

In summary, our results support the conclusion that TTVr improves functional capacity and QoL in patients with heart failure. Moreover, it seems to do so without regard to the presence of RVR, even though there might be indications of at least some impact of RVR on right heart function after TTVr.

### Mid-term clinical outcomes

Despite the refinement of functional capacity and QoL, the association with mid-term survival is still largely unknown in the context of TTVr. The only randomized trial so far in this field showed no survival benefit after edge-to-edge TR repair [10]. Compared with this study, our overall observed rate of all-cause mortality at the 12-month follow-up was higher (19%), which can probably relate to a sicker population and higher prevalence of severe comorbidities (more patients with diabetes, kidney disease, chronic obstructive lung disease, and NYHA class III/IV for instance). On the contrary, other early experience observational studies had similar one-year outcomes after TTVr and TMVr [38, 39].

Considering the role of maladaptive RVR in unfavorable clinical outcomes, concerns have been raised in TMVr [40]. Similar results were observed in patients who underwent TV surgery, as mentioned earlier [13]. Additionally, we observed that patients with RVR showed higher prevalence of the combined endpoint of one-year mortality and HF hospitalization. Most importantly, RVR was independently associated with all-cause mortality and the combined endpoint of mortality or rehospitalization after TTVr. Consequently, even though our data suggest an improvement in functional capacity and quality of life regardless of TR etiology already after 1 month, mid-term clinical outcomes and survival in particular seem to be negatively affected by the presence of preprocedural RVR. This hypothesis-generating finding should be further analyzed in larger controlled studies to confirm the causality of RVR in the prognosis of patients after TTVr.

### Limitations

Several limitations should be acknowledged. First, this is an observational single-center study, and our results are hypothesis-generating. Second, imaging quality was not high enough in all patients for 3D image acquisition; thus, more elaborate echo measures on RV anatomy and function are lacking. Third, there was no adjudication committee for the reported clinical outcomes. Fourth, differentiation of TR phenotype did not lie in the scope of this manuscript as our goal was to provide a hands-on parameter for risk assessment in everyday clinical practice. Nevertheless, further research in this direction should be promoted to fully elucidate the complex pathophysiology and associated risk in patients with TR. Finally, we analyzed not only one but several devices which have been implanted in consecutive patients. However, outcomes between devices were not different, and additionally reporting real-world outcomes should be considered as a strength of this study.

## Conclusion

TTVr resulted in significant QoL improvement already after 1 month, irrespective of RVR. Patients with RVR showed higher 1-year mortality and HF hospitalization rates; RVR was independently associated with all-cause mortality and the combined endpoint of mortality or rehospitalization after TTVr.

## Supplementary Information

Below is the link to the electronic supplementary material.

Supplementary file1 (DOCX 18 KB)

## Abbreviations

6 MWD: Six-minute walking distance
CI: Confidence interval
EROA: Effective regurgitant orifice area
FAC: Fractional area change
HF: Heart failure
HR: Hazard ratio
IQR: Interquartile range
MLHFQ: Minnesota Living with Heart Failure Questionnaire
NYHA: New York Heart Association
QoL: Quality of life
RV: Right ventricle/right ventricular
RVR: Right ventricular remodeling
SF-36: 36-Item Short Form Health Survey
TAPSE: Tricuspid annular plane systolic excursion
TR: Tricuspid regurgitation
TTVr: Transcatheter tricuspid valve repair
TV: Tricuspid valve
VC: Vena contracta

## Declarations

This manuscript has not been published and is not under consideration for publication elsewhere.
