# Supplementary material for: Role of preexisting right ventricular remodeling in symptoms and prognosis after transcatheter tricuspid valve repair
Source: Clin Res Cardiol. 2024 Mar 6;114(2):187–202. doi: 10.1007/s00392-024-02428-z (PMC11839808; doi:10.1007/s00392-024-02428-z)
Supplement: Supplementary file 1 — Supplementary file1 (DOCX 18 KB) [file 392_2024_2428_MOESM1_ESM.docx]

| Supplemental File. **Differences between TTVr devices** | | |  | | |
| --- | --- | --- | --- | --- | --- |
|  | **Total**  **(N=223)** | **Edge-to-Edge**  **(N=136)** | | **Direct Annuloplasty (N=87)** | **P-value**  **edge-to-edge**  **vs.**  **Direct Annuloplasty** |
| Technical Success | 209 (94) | 128 (94) | | 81 (93) | 0.761 |
| TR grade at discharge  I  II  III  IV  V | 58 (26)  86 (39)  62 (28)  12 (5)  5 (2) | 39 (29)  56 (41)  35 (26)  3 (2)  3 (2) | | 19 (22)  30 (35)  27 (31)  9 (10)  2 (2) | 0.071 |
| Death during TTVr | 5 (2) | 2 (1.5) | | 3 (3.5) | 0.331 |
| Myocardial Infarct after TTVr | 1 (0.5) | 0 (0) | | 1 (1) | 0.210 |
| Neurological Event after TTVr  Ischemic  Hemorrhagic | 2 (1)  1 (0.5) | 0 (0)  1 (1) | | 2 (2)  0 (0) | 0.286 |
| Bleeding after TTVr  Minor  Major  Extensive  Life-threatening  Lethal | 4 (2)  19 (8)  2 (1)  3 (1)  0 (0) | 0 (0)  11 (8)  1 (1)  2 (2)  0 (0) | | 4 (5)  8 (9)  1 (1)  1 (1)  0 (0) | 0.154 |
| Arrhythmia after TTVr  New atrial fibrillation  Ventricular tachycardia/ fibrillation | 2 (1)  0 (0) | 1 (1)  0 (0) | | 1 (1)  0 (0) | 0.749 |
| Acute kidney injury after TTVr | 15 (7) | 6 (4) | | 9 (10) | 0.086 |
| Device detachment | 3 (1) | 1 (1) | | 2 (2) | 0.323 |
| Emergent heart surgery after TTVr | 1 (0.5) | 0 (0) | | 1 (0) | 0.210 |
| Values are n (%).Technical success was defined as successful device implantation without conversion to emergent TV surgery or re-intervention, absence of mortality, and successful deployment and correct positioning of the device.  Bleeding was defined as minor = any overt bleeding that does not meet criteria for major, major = overt blood loss with hemoglobin decrease of > 3g/dl, extensive = requiring transfusion of blood products, life-threatening = bleeding that causes hemodynamic compromise requiring specific treatment, lethal = patient expired. Acute kindey injury was defined as increase in creatinine by ≥ 150% of baseline creatinine. | | | | | |
